# An assessment of sex bias in neurodevelopmental disorders

**DOI:** 10.1186/s13073-015-0216-5

**Published:** 2015-08-27

**Authors:** Andrew Polyak, Jill A. Rosenfeld, Santhosh Girirajan

**Affiliations:** Department of Biochemistry and Molecular Biology, The Pennsylvania State University, University Park, PA 16802 USA; Signature Genomic Laboratories, PerkinElmer, Inc., Spokane, WA 99207 USA; The Huck Institutes of the Life Sciences, The Pennsylvania State University, University Park, PA 16802 USA; Department of Anthropology, The Pennsylvania State University, 205A Huck Life Sciences Building, University Park, PA 16802 USA; Present address: Department of Molecular and Human Genetics, Baylor College of Medicine, Houston, TX 77030 USA

## Abstract

**Background:**

Neurodevelopmental disorders such as autism and intellectual disability have a sex bias skewed towards boys; however, systematic assessment of this bias is complicated by the presence of significant genetic and phenotypic heterogeneity of these disorders.

**Methods:**

To assess the extent and characteristics of sex bias, we analyzed the frequency of comorbid features, the magnitude of genetic load, and the existence of family history within 32,155 individuals ascertained clinically for autism or intellectual disability/developmental delay (ID/DD), including a subset of 8,373 individuals carrying rare copy-number variants (CNVs).

**Results:**

We find that girls were more likely than boys to show comorbid features within both autism (*P* = 2.9 × 10^−6^, OR = 1.34) and ID/DD (*P* = 7.2 × 10^−4^, OR = 1.08) cohorts. The frequency of comorbid features in ID/DD was higher in boys (1q21.1 deletion, 15q11.2q13.1 duplication) or girls (15q13.3 deletion, 16p11.2 deletion) carrying specific CNVs associated with variable expressivity while such differences were the smallest for syndromic CNVs (Smith-Magenis syndrome, DiGeorge syndrome). The extent of the male sex bias also varied according to the specific comorbid feature, being most extreme for autism with psychiatric comorbidities and least extreme for autism comorbid with epilepsy. The sex ratio was also specific to certain CNVs, from an 8:1 male:female ratio observed among autistic individuals carrying the 22q11.2 duplication to 1.3:1 male:female ratio in those carrying the 16p11.2 deletion. Girls carried a higher burden of large CNVs compared to boys for autism or ID/DD, and this difference diminished when severe comorbidities were considered. Affected boys showed a higher frequency of neuropsychiatric family histories such as autism (*P* = 0.01) or specific learning disability (*P* = 0.03), while affected girls showed a higher frequency of developmental family histories such as growth abnormalities (*P* = 0.02).

**Conclusions:**

The sex bias within neurodevelopmental disorders is influenced by the presence of specific comorbidities, specific CNVs, mutational burden, and pre-existing family history of neurodevelopmental phenotypes.

**Electronic supplementary material:**

The online version of this article (doi:10.1186/s13073-015-0216-5) contains supplementary material, which is available to authorized users.

## Background

Neurodevelopmental disorders such as autism and intellectual disability/developmental delay (ID/DD) are associated with a sex bias, with the diagnosis skewing towards boys compared to girls. For example, a male:female ratio of 2:1 exists among individuals with ID/DD [1, 2] and a 4:1 ratio for individuals with autism diagnoses [3, 4]. Emerging evidence suggests a female protective model [5, 6] as an explanation for the lower number of girls affected with these disorders. However, systematic assessment of sex bias in relation to neurodevelopmental disorders has been complicated by several factors. First, neurodevelopmental disorders are often associated with extensive phenotypic heterogeneity. While most studies assign individuals into one broader-yet-distinct disease nosology, such as autism or ID/DD disorders, a range of developmental and behavioral phenotypes are comorbid to a large extent in these cohorts [7]. For example, a comorbidity of intellectual disability has been observed in as high as 70 % of individuals diagnosed with autism [4, 8, 9]. Similarly, a comorbidity of epilepsy has been documented in 30 % to 40 % of individuals with autism [10, 11]. These high rates of comorbidity were not limited to autism alone. In fact, 6 % to 50 % of individuals with epilepsy were reported to also have some psychiatric disorder [12] and 28 % to 40 % of individuals with intellectual disability were reported to have autistic features [9, 13].

Second, hundreds of genes and genomic regions have been identified for each of these neurodevelopmental disorders from studies of copy number variants (CNVs) and exome sequencing, suggesting significant genetic heterogeneity [14–17]. For example, CNVs including 7q11.23 duplication [18–20], 16p11.2 deletion [21–23], 17q12 deletion [24–26], 15q13.3 deletion [27–29], 22q11.2 deletion [30–32], and gene disruptive mutations in *CHD2* [33–35] and *SYNGAP1* [34–37] have all been significantly associated with ID/DD, autism, and schizophrenia phenotypes from independent cohort studies. Further, these genetic factors can compound to create severe or variable presentations [38, 39]. In fact, the CNV load as assessed by the frequency and size of rare CNVs in an individual has been correlated with a range of distinct disorders including dyslexia, bipolar disorder, schizophrenia, autism, and ID/DD [40–42].

Third, there has been a pervasive existence of family history of neuropsychiatric phenotypes reported within these disorders which may explain why affected individuals present with certain features over others [43–47]. In one recent example, phenotypic manifestations in individuals carrying *de novo* CNVs associated with variable expressivity correlated with parental phenotypes; parents with lower IQ scores were more likely to have children with a diagnosis of ID/DD and parents with higher social responsive scores (SRS) were more likely to have children with a diagnosis of autism [48, 49]. Other studies have also reported that parents of children with autism more frequently manifest autistic, schizophrenic, or bipolar features than parents of typically developing children [45, 50]. When a family history of other affected individuals exist, it is known that the sex of the affected individual will impact recurrence risk and higher risks associated with male probands, suggesting that girls may require a higher familial etiologic load to manifest neurodevelopmental phenotypes [51–54].

We hypothesized that these factors influence, to different extents, the sex bias present in neurodevelopmental disorders. We therefore assessed sex bias in relation to the presence of comorbid features, CNV load, and family history of developmental and behavioral features among 32,155 individuals with autism and ID/DD features who were referred for genetic testing by clinicians. Our results support a model where the genetic liability for manifestation of neurodevelopmental phenotypes exists at different thresholds in boys and girls. These thresholds can be altered by the frequency and type of comorbid features, presence of disease-associated deletions and duplications, and a family history of related phenotypes.

## Methods

### Clinical data

**Fig. 1:**
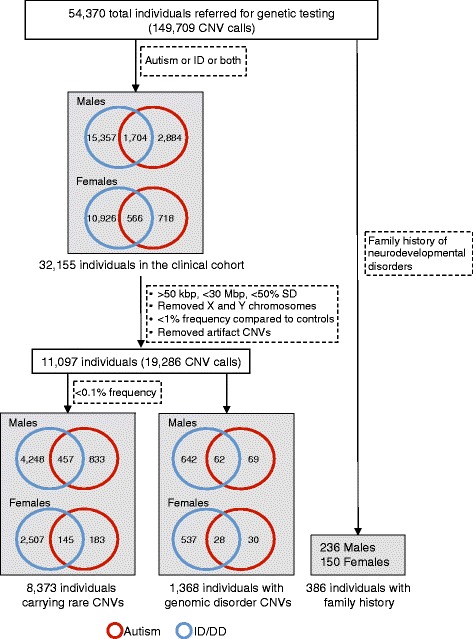
Schematic of cohorts derived from the clinical testing population. The schematic shows datasets derived from the Signature Genomics clinical testing population including the filtering of CNV calls to remove false-positive detection. Note that CNVs were removed if there was a >10 % overlap with a list of artifact CNV calls (Additional file 1: Table S2). Rare CNVs were derived after removing the CNV calls greater than 0.1 % frequency in a control cohort (>8/8,329 controls)

We analyzed clinical and CNV data from 54,370 individuals referred by clinicians to Signature Genomic Laboratories, LLC, for clinical testing from over 40 referral sites primarily in the United States and Canada (Fig. 1). Of these, 32,155 individuals showed features of autism or ID/DD. In this study, only de-identified phenotypic (case histories and clinical information) and CNV data were used (exempt from IRB review) and research conformed to the Helsinki Declaration. The phenotypic data consisted of diagnostic indications, age, and sex information reported through requisition forms by geneticists, pediatricians, and neurologists who have had direct contact with the patient. Patient indications included features of autism and/or ID/DD with or without comorbidities (or co-occurring features) such as epilepsy, speech, motor and language deficits, behavioral and psychiatric issues including schizophrenia and bipolar disorder, and other congenital malformations including cardiac defects, renal and genitourinary abnormalities, and craniofacial and skeletal features.

We curated a list of more than 75 non-redundant keywords corresponding to diagnostic terms from physician-reported clinical indications (Additional file 1: Table S1). In order to uniformly assess the frequency of phenotypes, we binned the diagnostic terms into seven broadly defined categories similar to the US Department of Education disability categories in special education according to the Individuals with Disabilities Education Act (IDEA) [55]. The seven phenotypic categories are autism, ID/DD, other health impairments (OHI), psychiatric disorders, behavioral disorders, speech and language impairments, and specific learning disabilities (Additional file 1: Table S1). For example, keywords such as ‘autism’ or ‘PDD’ were binned into the autism category, while ‘cognitive deficit’ or ‘mental retardation’ went into the ID/DD category, and ‘epilepsy’, ‘ADHD’, or ‘cardiac’ were binned into a broader OHI category). The OHI category, as defined previously [55], consisted of developmental and behavioral phenotypes that did not fit into any of the other categories. This category included growth abnormalities, dysmorphic features, cardiac malformations, epilepsy, attention deficit disorders, other CNS malformations, and other congenital malformations. Frequencies of comorbid features were assessed from all individuals with autism alone (n = 3,602), ID/DD alone (n = 26,283), both autism and ID/DD (n = 2,270), from clinics (n = 32,155), from the subset with rare CNVs (total n = 8,373, autism = 1,016, ID/DD = 6,755, both = 602), and from the subset carrying a CNV associated with genomic disorders (n = 1,368), and male and female subsets, separately (Fig. 1). Individuals with autism or ID also manifesting more than one comorbid feature were categorized into ‘multiple features’ or ‘multiple OHI features’ categories. Individuals with autism and ID/DD were included in the analysis of both autism and ID/DD cohorts.

We were also able to manually extract family history of one or more phenotypes from 386 affected individuals. Frequency of each type of family history relative to all cases with a family history was then calculated for boys and girls. We created a matrix by using the number of individuals with a specific indication and a specific family history as the numerator and the number of individuals with that indication with any positive family history as the denominator. Family histories were binned into 12 phenotypic categories, which can be placed into two broad groups based on the age-specific prevalence of these phenotypes (Additional file 1: Figure S1). Family history of features present at younger ages such as ID/DD, dysmorphic features, other congenital malformations, growth abnormalities, and cardiac malformations were binned into ID/DD and multiple congenital anomalies (MCA) disorders. Family history of features present in older children such as epilepsy, other CNS malformations, autism, psychiatric disorders, attention deficit disorders, speech and language impairments, and specific learning disabilities were binned into neuropsychiatric/behavioral disorders. To reduce overlap of frequently co-occurring features such as ID/DD, autism, and epilepsy, certain filters were also applied to both clinical indications and family history information. Then, we separately analyzed commonly occurring pairs of comorbid features including autism with ID/DD and ID/DD with epilepsy.

### CNV data

CNVs were identified and analyzed using whole genome oligonucleotide microarrays (customized SignatureChipOS v2 and v3 with 135,000 probes from Roche NimbleGen, Madison, WI, USA and customized SignatureChipOS v1 and v4 with 105,000 probes from Agilent Technologies, Santa Clara, CA, USA) and validated by fluorescence *in situ* hybridization as described previously [30, 56, 57]. CNV calls were subjected to quality-control filtering to remove false positive and non-specific detections, technical artifacts, calls due to reference CNVs, and variants embedded within complex segmental duplications (Fig. 1). Rare CNVs were filtered to include only those ranging between 50 kbp and 30 Mbp and occurring at <0.1 % frequency compared to 8,329 controls [30, 56]. Further filtering was applied to include only those CNVs with <50 % overlap with segmental duplications and <10 % overlap with a list of CNV artifacts curated from previous studies (Additional file 1: Table S2). Due to lack of control data on sex chromosomes, only autosomal CNVs were considered. After quality control, 8,373 individuals with features of autism and/or ID/DD carrying rare CNVs were available for analysis. This group included recently published individuals with CNVs mapping within the 72 chromosomal regions associated with genomic disorders [56]; their phenotypic data were also included for this analysis. The genomic disorder CNVs were classified into those that are mostly *de novo* in occurrence and resulting in a typical constellation of clinical features (syndromes) and those that are often inherited and associated with various neurodevelopmental phenotypes including autism, schizophrenia, ID/DD, and epilepsy (variable expressivity) as described previously [56]. For replication of results obtained from the Signature cohort, we also utilized CNV data from individuals with autism from two independently ascertained cohorts: the Simons Simplex Collection (1,124 children) [19] and the Childhood Autism Risks from Genetics and Environment (CHARGE) study (272 children with autism and 242 typically developing controls) [42].

CNV burden was calculated as the population frequency of the largest CNV and plotted as a survivor function [58]. The population frequencies of the largest CNV at a given size threshold (≥1 Mbp, ≥1.5 Mbp, ≥2 Mbp) were compared with different combinations of comorbid features associated with autism or ID/DD disorders using Fisher’s exact test. One-tailed *P* values were used to determine significance for all statistical tests of comparison. A list of all rare CNV calls analyzed in this study is given in Additional file 1: Table S3. Rare CNV data used in this study were also deposited into dbVar (accession number: nstd113). Comprehensive tables including all statistical analyses (one-tailed and two-tailed *P* values) and their corresponding Bonferroni multiple testing corrections are included as Additional file 1: Tables S4-S8.

## Results

We analyzed clinical and CNV data from 54,370 individuals that were referred to Signature Genomic Laboratories for genetic testing by array CGH. Using keyword searches of physician-reported clinical indications, we identified 32,155 individuals with features of autism or ID/DD with or without comorbid features. Figure 1 shows a breakdown of the number of boys and girls with features of autism (n = 5,872) or ID/DD (n = 28,553), with 2,270 individuals manifesting both autism and ID/DD. From this cohort, 1,368 individuals carried rare CNVs associated with genomic disorders that were either syndromic or variable in clinical presentation [56], and 8,373 individuals carried rare CNVs seen in less than 0.1 % (<8/8,329) of the control population.

### Comorbid features in boys and girls with autism or ID/DD disorders

**Fig. 2:**
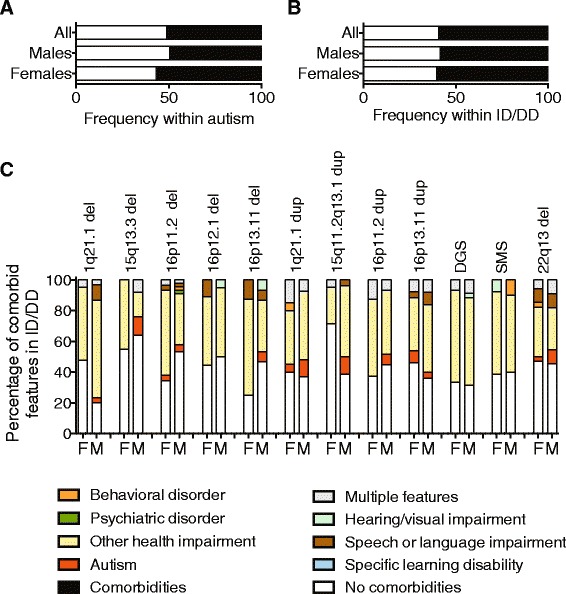
Comorbidity within autism and ID/DD. The frequency of comorbid features within (**a**) all cases with autism (n = 5,872), boys (n = 4,588) and girls (n = 1,284) with autism, and within (**b**) all individuals with ID/DD (n = 28,553), boys (n = 17,061) and girls (n = 11,492) with ID/DD is shown. The frequency of comorbid features within (**c**) girls (F) and boys (M) is shown for a representative set of genomic disorders. Additional file 1: Table S8 shows data for all CNVs with sample sizes >10. Due to a limited sample size within the genomic disorder cohort, only ID/DD can be shown. Samples sizes for the CNVs are: 1q21.1 del: M = 30, F = 21; 15q13.3 del: M = 25, F = 20; 16p11.2 del: M = 45, F = 29; 16p12.1 del: M = 20, F = 9; 16p13.11 del: M = 25, F = 26; 1q21.1 dup: M = 27, F = 20; 15q11.2q13.1 dup (Prader-Willi region dup): M = 26, F = 21; 16p11.2 dup: M = 29, F = 16; 16p13.11 dup: M = 25, F = 26; 22q11.2 del (DiGeorge Syndrome): M = 35, F = 30; Smith Magenis Syndrome (SMS): M = 10, F = 13; 22q13 del: M = 11, F = 34

Overall, comorbid features existed in 51 % (3,004/5,872) of individuals with autism and 59 % (16,902/28,553) of individuals with ID/DD (Fig. 2a, b). The total frequency of all comorbid features was higher among girls compared to boys within cohorts manifesting autism (*P* = 2.94 × 10^−6^, OR = 1.34, 95 % CI = 1.18-1.52) or ID/DD (*P* = 7.15 × 10^−4^, OR = 1.08, 95 % CI = 1.03-1.14) features (Additional file 1: Tables S4 and S5). This trend was also observed when a subset of individuals with rare CNVs (<0.1 % population frequency) was considered. We also analyzed the frequency of specific comorbid features binned under the OHI category including epilepsy, cardiac malformation, growth abnormalities, dysmorphic features, attention-deficit disorders, other CNS malformation, and other congenital malformation (Additional file 1: Table S1). Among individuals with any OHI comorbidity, girls with autism (*P* = 0.01, OR = 1.64, 95 % CI = 1.07–2.50) or ID/DD (*P* = 0.009, OR = 1.14, 95 % CI = 1.02–1.27) were more likely to show comorbid features of epilepsy compared to boys (Additional file 1: Figure S2, Tables S6, S7). Among individuals with ID/DD and any OHI comorbidity, boys were more likely to show comorbidity of dysmorphic features (*P* = 0.004, OR = 1.10, 95 % CI = 1.02–1.18) and attention deficit disorders (*P* = 0.0006, OR = 1.95, 95 % CI = 1.29–3.03) compared to girls (Additional file 1: Table S7).

We analyzed comorbidity rates within a subset of 1,368 individuals carrying specific deletions and duplications associated with genomic disorders and compared the frequency of comorbid features in boys and girls. Because we restricted our analysis to sample sizes ≥10, we were only able to assess the comorbidity frequencies for CNVs within individuals with ID/DD but not with autism. Among individuals with ID/DD (Fig. 2c, Additional file 1: Table S8), trends toward a higher frequency of comorbid features were observed in girls or boys for certain CNVs. These differences were larger for CNVs associated with variable expressivity, and the smallest differences were seen for CNVs associated with syndromes such as DiGeorge syndrome, Phelan-McDermid syndrome, and Smith-Magenis syndrome.

### Sex ratio of individuals with autism or ID/DD

**Fig. 3:**
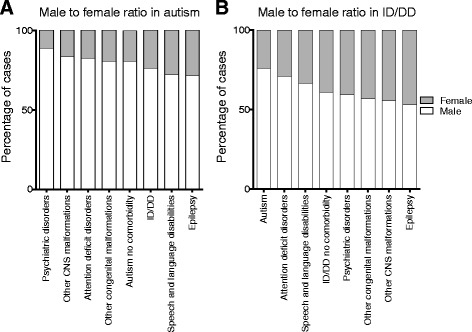
Sex ratio for specific comorbidities in individuals with autism and ID/DD. The male:female ratio of individuals manifesting (**a**) autism, and (**b**) ID/DD, with specific comorbidities is shown. For this analysis, the other congenital malformations category also included growth abnormalities, kidney malformations, cardiac malformations, and dysmorphic features. Sample sizes (n) for each combination of comorbid features (with autism or ID/DD) is provided in Additional file 1: Table S9

We sought to assess the male:female ratio of individuals with autism and ID/DD who were also showing specific comorbidities (Fig. 3). First, the sex ratio of autism without comorbidities was 4.2:1, similar to recent epidemiological reports [3]. Interestingly, we found that the sex ratios of individuals with autism or ID/DD varied based on specific comorbid clinical features. In fact, while an 8:1 sex ratio existed among individuals with autism also manifesting psychiatric features, a 3.2:1 sex ratio was observed among autistic individuals manifesting ID/DD, and a 2.5:1 sex ratio for those also manifesting epilepsy (Additional file 1: Table S9). Similarly, when compared to the 1.6:1 ratio for ID/DD without comorbidities, the sex ratio increased to 3.2:1 in individuals with autism comorbidity and decreased to 1.1:1 in individuals manifesting ID/DD with features of epilepsy.

**Fig. 4:**
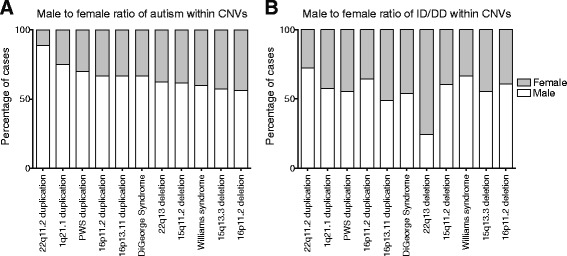
Sex ratio for individuals with autism or ID/DD also carrying specific CNVs. The male:female ratio of individuals carrying specific deletions and duplications also manifesting features of (**a**) autism and (**b**) ID/DD. Sample sizes (n) for each CNV is provided in Additional file 1: Table S10

Sex ratio also varied when the number of boys and girls carrying specific CNVs were assessed (Fig. 4, Additional file 1: Table S10). For example, among individuals with autism, a male:female ratio of 3:1 was observed for 1q21.1 duplication, 2.3:1 for 15q11.2q13.1 duplication, 1.6:1 for 15q11.2 deletion, and 1.3:1 for 16p11.2 deletion. Further, among individuals with ID/DD, a male:female ratio of 2.6:1 was observed for 22q11.2 duplication, 1.6:1 for 16p11.2 deletion, and 0.3:1 for 22q13 deletion. While bias in clinical ascertainment can contribute to these observations, our results suggest that the spectrum of autism or ID/DD in boys and girls is contingent upon the presence of specific comorbid features and rare CNVs of varying clinical significance.

### CNV burden among boys and girls with autism, ID/DD, and comorbid phenotypes

**Fig. 5:**
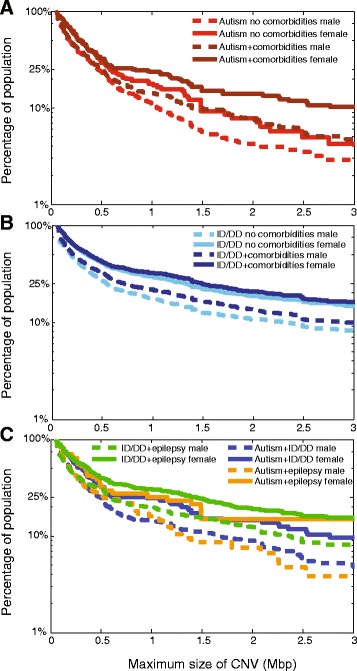
CNV burden in autism and ID/DD cohorts. Population frequency of the largest CNV in individuals with autism, ID/DD, epilepsy, and/or other comorbidities is shown. Using the rare CNV cohort, survivor functions were generated for the population frequency carrying a CNV larger than a given size. CNV burden plots are shown for: (**a**) boys (dashed lines) and girls (solid lines) with autism with or without any comorbidities; (**b**) boys (dashed lines) and girls (solid lines) with ID/DD with or without any comorbidities; (**c**) boys (dashed lines) and girls (solid lines) with autism with ID, autism with epilepsy, and ID with epilepsy are shown. Full statistical analysis including sample sizes (n) is provided in Additional file 1: Table S11

We explored a sex bias for rare CNV burden among individuals with autism or ID/DD with and without comorbid phenotypes (Fig. 5, Additional file 1: Table S11). Overall, when all comorbidities were considered separately or in aggregate, girls showed a significantly increased large CNV burden compared to boys for a primary diagnosis of autism or ID/DD (Fig. 5a-c). Further, when individuals manifesting autism or ID/DD without any comorbidities were compared, girls showed a higher burden for large CNVs compared to boys (Fig. 5a, b). Interestingly, this difference in CNV burden between boys and girls seemed to decrease in magnitude when individuals ascertained with ID/DD were considered (Fig. 5b). These observations were also replicated by a reanalysis of previously published CNV data from independently ascertained autism cohorts from the Simons Simplex Collection [19] and the CHARGE study [42] (Additional file 1: Figure S3A, B, Table S12). A significantly higher CNV burden was also observed for girls compared to boys (Mann Whitney test, one-tailed *P* = 0.009) manifesting autism with or without ID/DD when total base pairs of *de novo* CNV per person in these independently ascertained cohorts were considered (Additional file 1: Figure S3C).

### Assessment of family history of neurodevelopmental phenotypes in boys and girls

**Fig. 6:**
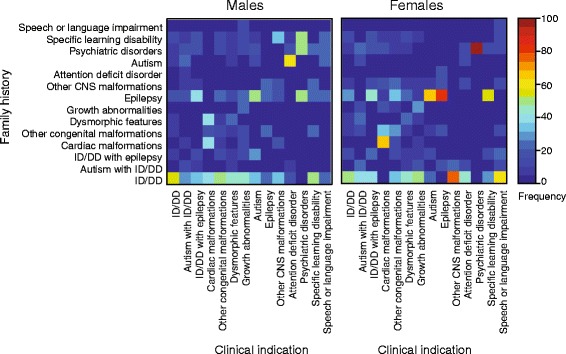
Family history among individuals with neurodevelopmental phenotypes. Matrices were generated showing percentage of individuals with a clinical indication and a specific family history among all individuals with that specific indication and any family history: boys (left, n = 236) and girls (right, n = 150). Family history is represented on the Y-axis, and the clinical indication of the proband is on the X-axis. The frequency of family history is depicted as a range of colors. A graphical description of the comparisons made is provided in Additional file 1: Figure S4

From the 386 individuals with neurodevelopmental phenotypes for whom family history information was available, a frequency matrix of family history and clinical indications grouped into 12 phenotypic categories was generated. Frequencies were calculated from the number of individuals with a specific indication and a specific family history as the numerator and the total number of individuals with that indication with any family history as the denominator (Fig. 6, Additional file 1: Figure S4). A family history of ID/DD tended to exist consistently in all clinical indications. Although no sex difference was observed when all family histories were considered in aggregate (*P* = 0.48), significant differences existed within specific family history phenotypes. Of all affected individuals with a family history of autism (Student’s *t* test, one-tailed *P* = 0.01) and specific learning disability (Student’s *t* test, one-tailed *P* = 0.03), we found a higher frequency of boys compared to girls. In contrast, we observed a higher frequency of girls than boys when individuals with a family history of growth abnormalities were considered (Student’s *t* test, one-tailed *P* = 0.02). We were then able to categorize the 12 phenotypes into two broad groups of ID/DD/MCA disorders and neuropsychiatric/behavioral disorders based on the age-specific prevalence within the cohort (Additional file 1: Figure S1). Among individuals with a family history of a broader ID/DD/MCA disorders, no differences in the frequency of boys compared to girls with indications of ID/DD/MCA were observed (Mann Whitney test, *P* = 0.13). However, we observed a higher frequency of boys manifesting neuropsychiatric/behavioral disorders compared to girls when all individuals with a family history of neuropsychiatric/behavioral disorders were evaluated (Mann Whitney test, *P* = 0.03).

## Discussion

We sought to dissect the various factors affecting sex bias in autism and intellectual disability disorders. This study is the first to connect clinical data including diagnostic indications and family history with genomic data on CNVs. Ideally, standardized diagnostic measures and deep phenotyping would be useful in the ascertainment of individuals with neurodevelopmental disorders. However, such measures are not always available, especially within a large clinically heterogeneous population such as the Signature Genomics cohort. Instead, we provide a realistic assessment of how the documented sex bias extends to other facets of heterogeneity in the diagnosis of neurodevelopmental disorders.

Several themes have emerged from our study. First, the pervasive presence of comorbid features among affected individuals was evident in our dataset, existing in about half of individuals ascertained for autism or ID/DD. We found that these comorbid features manifested at different frequencies among boys and girls, with girls as a whole more likely to have comorbidities. Within specific categories of comorbidities, girls were more likely than boys to manifest epilepsy with either autism or ID/DD, consistent with previous reports [59–61]. These results suggest that while autism and ID/DD disorders have a sex bias skewed towards boys, girls are more likely to be diagnosed when they have additional comorbid features. Interestingly, the sex difference in the presence of comorbid features also varied across individuals carrying specific CNVs. Differences were most notable for individuals carrying CNVs associated with variable phenotypes, with skewing toward either sex, while in individuals with DiGeorge syndrome and Smith-Magenis syndrome, where individuals typically manifest a more fixed constellation of features, the frequency of comorbid features was invariable among the sexes.

Second, the sex bias was not uniform across specific comorbid features and specific CNVs. In fact, while a 4.2:1 male:female ratio existed for autism without comorbidities, a sex ratio shift toward girls (3.2:1) was observed for autism with ID/DD and toward boys (8:1) for autism with psychiatric disorders. Our results are also supported by previous studies that show a sex ratio of 6:1 [62] in high-functioning individuals with autism and 2:1 within individuals with autism manifesting intellectual disability [63]. For those individuals ascertained for ID/DD, while there was a 1.6:1 male:female ratio for those with no comorbidities, we found a 1.1:1 ratio for individuals also manifesting epilepsy. The sex ratio of autism and ID/DD also corresponded to specific CNVs. For example, while individuals with autism carrying the 22q11.2 duplication had an 8:1 male:female ratio, those carrying a 15q13.3 deletion or 16p11.2 deletion showed a 1.3:1 sex ratio. Similarly, individuals with ID/DD and carrying 22q11.2 duplication showed a male:female ratio of 2.6:1, while those carrying DiGeorge syndrome deletion or 15q11.2q13.1 (PWS region) duplication showed a 1.2:1 sex ratio. Our analysis suggests that girls, while exhibiting more comorbidities than boys, are ascertained at a closer frequency to their male counterparts when they are more severely affected.

Third, the frequency of large CNVs, or CNV burden, also corresponded to sex. Girls showed a higher CNV burden than boys for autism alone or autism with comorbid features. Interestingly, the CNV burden among girls with autism with no comorbidities was similar to that in boys manifesting autism with comorbidities (Fig. 5). This is consistent with the recently reported female protective model for neurodevelopmental disorders [6]. While a male to female difference in CNV burden was observed among individuals manifesting features of ID/DD without comorbidities and ID/DD with comorbidities, this difference in CNV burden diminished in the latter cohort (Fig. 5c). These results suggest that the sex difference in CNV burden dissolves when applied to severely affected subsets.

Finally, the existence of positive family histories among individuals ascertained for neurodevelopmental phenotypes enabled us to further dissect the complex nature of these disorders. Although there was a lack of statistical power to derive significant associations for all phenotypes of varying severity analyzed for presenting indications and family history, we found that affected boys predominantly had neuropsychiatric family histories such as autism or specific learning disability, whereas affected girls had developmental disorders in their family histories such as growth abnormalities. A lack of statistical power made any assessment of genetic liability within this subset of individuals difficult. It is likely that individuals with a family history of affected individuals are more predisposed to a condition due to an increased genetic liability within their family, and our results suggest that sex also affects the likelihood of a child in these families having a neurodevelopmental disorder [52].

## Conclusions

From our results, it is clear that the sex bias is not limited to the initial diagnosis of autism or ID/DD, but affects the extent of heterogeneity. We observed variations in male:female ratios based on comorbid features, presence of CNVs, and family history, but these ratios tend to diminish when applied to severely affected individuals. Future studies of the phenotypic heterogeneity surrounding neurodevelopmental disorders should take sex bias into account.

## Additional file

Additional file 1: Table S1. Keywords used to bin phenotypic indications from clinical referrals into phenotypic categories. **Table S2.** A list of CNV artifacts used for removing false positive calls is provided as an Excel file. **Table S3.** A list of all rare CNV calls is provided as an Excel file. **Table S4.** Frequency of comorbid features among boys and girls with autism in the (clinical and rare CNV) study cohort. **Table S5.** Frequency of comorbid features among boys and girls with ID/DD in the (clinical and rare CNV) study cohort. **Table S6.** Frequency of comorbid specific OHI features among boys and girls with autism in the (clinical and rare CNV) study cohort. **Table S7.** Frequency of comorbid specific OHI features among boys and girls with ID/DD in the (clinical and rare CNV) study cohort. **Table S8.** Frequency of comorbid features in boys and girls carrying specific CNVs associated with genomic disorders. **Table S9.** Ratio of boys to girls in individuals with autism and ID/DD and showing specific comorbid features. **Table S10.** Ratio of boys to girls in individuals with autism or ID/DD and carrying specific CNVs. **Table S11.** Comparison of rare CNV load for specific combination of comorbid features in autism and ID/DD. **Table S12.** Comparison of CNV burden between boys and girls ascertained for autism or controls in two independent cohort studies. **Figure S1.** Age specific prevalence of ID/DD/MCA and neuropsychiatric/behavioral features within the clinical dataset. **Figure S2.** Frequency of comorbid features within the OHI category for clinical and rare CNV cohorts. **Figure S3.** Replication of CNV burden results in two independent cohort studies. **Figure S4.** Explanation of the comparisons made in the family history matrices. (ZIP 1274 kb)

## Abbreviations

CNV: Copy number variant
ID/DD: Intellectual disability/developmental delay
MCA: Multiple congenital anomalies
OHI: Other health impairments
