# Supplementary material for: An assessment of sex bias in neurodevelopmental disorders
Source: Genome Med. 2015 Aug 27;7(1):94. doi: 10.1186/s13073-015-0216-5 (PMC4549901; doi:10.1186/s13073-015-0216-5)
Supplement: Additional file 1: Table S1. — Keywords used to bin phenotypic indications from clinical referrals into phenotypic categories. Table S2. A list of CNV artifacts used for removing false positive calls is provided as an Excel file. Table S3. A list of all rare CNV calls is provided as an Excel file. Table S4. Frequency of comorbid features among boys and girls with autism in the (clinical and rare CNV) study cohort. Table S5. Frequency of comorbid features among boys and girls with ID/DD in the (clinical and rare CNV) study cohort. Table S6. Frequency of comorbid specific OHI features among boys and girls with autism in the (clinical and rare CNV) study cohort. Table S7. Frequency of comorbid specific OHI features among boys and girls with ID/DD in the (clinical and rare CNV) study cohort. Table S8. Frequency of comorbid features in boys and girls carrying specific CNVs associated with genomic disorders. Table S9. Ratio of boys to girls in individuals with autism and ID/DD and showing specific comorbid features. Table S10. Ratio of boys to girls in individuals with autism or ID/DD and carrying specific CNVs. Table S11. Comparison of rare CNV load for specific combination of comorbid features in autism and ID/DD. Table S12. Comparison of CNV burden between boys and girls ascertained for autism or controls in two independent cohort studies. Figure S1. Age specific prevalence of ID/DD/MCA and neuropsychiatric/behavioral features within the clinical dataset. Figure S2. Frequency of comorbid features within the OHI category for clinical and rare CNV cohorts. Figure S3. Replication of CNV burden results in two independent cohort studies. Figure S4. Explanation of the comparisons made in the family history matrices. (ZIP 1274 kb) [file 13073_2015_216_MOESM1_ESM.zip › additional files/Polyak_Supplemental_REVISED4_FINAL.docx]

**Additional files**

An assessment of sex bias in neurodevelopmental disorders

Andrew Polyak^1^

E-mail: ajp5435@psu.edu

Jill A. Rosenfeld^2, 3^

E-mail: Jill.Mokry@bcm.edu

Santhosh Girirajan^1, 4, 5^

E-mail: sxg47@psu.edu

1. Department of Biochemistry and Molecular Biology, The Pennsylvania State University, University Park, PA 16802
2. Signature Genomic Laboratories, PerkinElmer, Inc., Spokane, WA 99207
3. Department of Molecular and Human Genetics, Baylor College of Medicine, Houston, TX 77030
4. The Huck Institutes of the Life Sciences, The Pennsylvania State University, University Park, PA 16802
5. Department of Anthropology, The Pennsylvania State University, University Park, PA 16802

Table of Contents

[1. Supplemental Tables 3](#_Toc424902518)

[Table S1: Keywords used to bin phenotypic indications from clinical referrals into phenotypic categories 3](#_Toc424902519)

[Table S2: A list of CNV artifacts used for removing false positive calls is provided as an Excel file. 4](#_Toc424902520)

[Table S3: List of all rare CNV calls analyzed in this study is provided as an Excel file. 4](#_Toc424902521)

[Table S4: Frequency of comorbid features among males and females with autism in the study cohort 4](#_Toc424902522)

[Table S5: Frequency of comorbid features among males and females with ID/DD in the study cohort 5](#_Toc424902523)

[Table S6: Frequency of comorbid specific OHI features among males and females with autism in the study cohort 6](#_Toc424902524)

[Table S7: Frequency of comorbid specific OHI features among males and females with ID/DD in the study cohort 7](#_Toc424902525)

[Table S8: Frequency of comorbid features among individuals carrying CNVs associated with genomic disorders in males and females 8](#_Toc424902526)

[Table S9: Ratio of males to females within the clinical cohort according to comorbidities 9](#_Toc424902527)

[Table S10: Ratio of males to females carrying specific CNVs affected with autism or ID/DD 10](#_Toc424902528)

[Table S11: Comparison of rare CNV load for comorbid features in autism and ID/DD 11](#_Toc424902529)

[Table S12: Comparison of CNV burden between males and females ascertained for autism or controls in two independent cohort studies 13](#_Toc424902530)

[2. Supplemental Figures 14](#_Toc424902531)

[Figure S1: Age specific prevalence of ID/DD/MCA (left) and neuropsychiatric/behavioral features 14](#_Toc424902532)

[Figure S2: Frequency of comorbid features within the OHI category for clinical and rare CNV cohorts 15](#_Toc424902533)

[Figure S3: Replication of CNV burden results in two independent cohort studies. 16](#_Toc424902534)

[Figure S4: Explanation of the comparisons made in the family history matrices 17](#_Toc424902535)

[REFERENCES 18](#_Toc424902536)

# 1. Supplemental Tables

## **Table S1**: Keywords used to bin phenotypic indications from clinical referrals into phenotypic categories

| **Category** | **Clinical indications noted in the physician requisition forms for genetic testing** |
| --- | --- |
| **Autism** | Autism, Asperger, Pervasive Developmental Delay (PDD) |
| **Intellectual disability/developmental delay (ID/DD)** | Mental Retardation (MR), Intellectual Disability (ID) , Developmental delay (DD), Cognitive Deficit |
| **Other health impairments (OHI)** | **Other CNS malformation** Paraplegia, Ataxia, Cerebral disorders, Coordination, Chiari malformation, Brain malformation, Dandy-walker syndrome, Periventricular disorder, Agenesis of callosum, Microgyria |
|  | **Growth abnormality** Failure to thrive, Hypotonia, Feeding difficulties, Myopathy, Weakness, Growth abnormality, Tall/short stature |
|  | **Dysmorphic feature** Synostosis, Frontal bossing, Cleft lip/palate, Skeletal malformation, Exostosis, Syndactyly, Microcephaly, Macrocephaly |
|  | **Cardiac malformation**  Cardiac, Vascular, Septal, Heart, Valve, Ventricular Septal Defect (VSD), Atrial Septal Defect (ASD), Tetralogy of Fallot (TOF), Patent Ductus Arteriosis (PDA), Congenital Heart Disease (CHD), aneurysm, aorta, coarctation, arterial |
|  | **Epilepsy**  Epilepsy, Encephalopathy, Seizure, Febrile, Convulsion, Spasm |
|  | **Attention deficit disorder**  Attention Deficit Disorder, Hyperactivity |
|  | **Other congenital malformation**  Holoprosencephaly, Multiple congenital, Heterotaxy, Hydrocephaly, Other malformations, Renal, Kidney, Polycystic, Multicystic, Horseshoe, Ureter, Urinary |
| **Psychiatric disorders** | Schizophrenia, Bipolar, Psychological, Paranoia |
| **Behavior disorders** | Behavior, Aggression, Anger, Sleep |
| **Speech or language impairments** | Language, Speech, Stutter, Voice impairment |
| **Specific learning disabilities** | Dyslexia, Learning |

Note that Hearing and Vision impairments (from IDEA definitions) are not shown here and not part of the seven categories (Methods) but included in the frequency calculations.

## Table S2: A list of CNV artifacts used for removing false positive calls is provided as an Excel file.

## Table S3: A list of all rare CNV calls is provided as an Excel file. CNV calls are also deposited in dbVar (nstd113).

## **Table S4**: Frequency of comorbid features among males and females with autism in the study cohort

| Clinical | Frequency in males (n=4,588) | Frequency in females (n=1,284) | 1 tail p-value | 1 tail corrected | 2 tail p-value | 2 tail corrected | Odds ratio | Lower 95% CI | Upper 95% CI |
| --- | --- | --- | --- | --- | --- | --- | --- | --- | --- |
| Autism no comorbidities | 0.50 | 0.43 | 2.9E-06 | 2.9E-05 | 5.4E-06 | 5.4E-05 | 1.34 | 1.18 | 1.52 |
| ID/DD | 0.23 | 0.26 | 0.02 | 0.24 | 0.04 | 0.44 | 1.16 | 1.00 | 1.34 |
| OHI | 0.11 | 0.11 | 0.45 | NA | 0.88 | NA | 1.02 | 0.83 | 1.25 |
| Hearing | 0.00218 | 0 | 0.08 | NA | 0.13 | NA | Inf | 0.63 | Inf |
| Vision | 0 | 0.001 | 0.22 | NA | 0.22 | NA | Inf | 0.09 | Inf |
| Psychiatric | 0.002 | 0.001 | 0.38 | NA | 0.69 | NA | 2.24 | 0.30 | 99.48 |
| Behavior | 0.003 | 0 | 0.04 | 0.40 | 0.09 | NA | Inf | 0.85 | Inf |
| Learning | 0.002 | 0.001 | 0.27 | NA | 0.47 | NA | 2.80 | 0.40 | 121.66 |
| Speech/language | 0.003 | 0.007 | 0.08 | NA | 0.09 | NA | 2.02 | 0.78 | 4.86 |
| Multiple features | 0.15 | 0.20 | 5.1E-05 | 5.1E-04 | 8.8E-05 | 8.8E-04 | 1.38 | 1.17 | 1.63 |
| Rare CNVs | (n=1,290) | (n=328) |  |  |  |  |  |  |  |
| Autism no comorbidities | 0.51 | 0.43 | 5.6E-03 | 0.06 | 0.01 | 0.11 | 1.38 | 1.07 | 1.78 |
| ID/DD | 0.21 | 0.22 | 0.32 | NA | 0.60 | NA | 1.08 | 0.79 | 1.46 |
| OHI | 0.12 | 0.11 | 0.33 | NA | 0.63 | NA | 1.12 | 0.75 | 1.69 |
| Hearing | 0.001 | 0 | 0.80 | NA | 1.00 | NA | Inf | 6.5E-03 | Inf |
| Vision | 0 | 0 | 1.00 | NA | 1.00 | NA | Inf | 0 | Inf |
| Psychiatric | 0.002 | 0 | 0.64 | NA | 1.00 | NA | Inf | 0.05 | Inf |
| Behavior | 0.004 | 0 | 0.32 | NA | 0.59 | NA | Inf | 0.23 | Inf |
| Learning | 0 | 0.003 | 0.20 | NA | 0.20 | NA | Inf | 0.10 | Inf |
| Speech/language | 0.004 | 0.003 | 0.65 | NA | 1.00 | NA | 1.27 | 0.14 | 60.36 |
| Multiple features | 0.15 | 0.23 | 3.6E-04 | 3.6E-03 | 6.3E-04 | 6.3E-03 | 1.71 | 1.25 | 2.33 |

Comparisons of frequency of specific comorbid features in males and females with autism in the clinical and rare CNV cohorts. **A graphical representation of autism with and without comorbidities is shown in Figure 2A.** Fisher’s exact test was used for statistical analysis. Bonferroni correction was applied (corrected p-values) by multiplying p-values by 10, the number of tests performed in each cohort.

## Table S5: Frequency of comorbid features among males and females with ID/DD in the study cohort

| Clinical | Frequency in males (n=17,061) | Frequency in females (n=11,492) | 1 tail p-value | 1 tail corrected | 2 tail p-value | 2 tail corrected | Odds ratio | Lower 95% CI | Upper 95% CI |
| --- | --- | --- | --- | --- | --- | --- | --- | --- | --- |
| ID/DD no comorbidities | 0.42 | 0.40 | 7.2E-04 | 7.2E-03 | 1.4E-03 | 0.01 | 1.08 | 1.03 | 1.14 |
| Autism | 0.06 | 0.03 | 1.4E-39 | 1.4E-38 | 2.6E-39 | 2.6E-38 | 2.23 | 1.96 | 2.54 |
| OHI | 0.43 | 0.50 | 5.8E-36 | 5.8E-35 | 1.1E-35 | 1.1E-34 | 1.35 | 1.29 | 1.42 |
| Hearing | 0.003 | 0.003 | 0.53 | NA | 1.00 | NA | 1.01 | 0.64 | 1.61 |
| Vision | 0.001 | 0.001 | 0.36 | NA | 0.61 | NA | 1.19 | 0.58 | 2.41 |
| Psychiatric | 0.001 | 0.001 | 0.55 | NA | 1.00 | NA | 1.02 | 0.43 | 2.34 |
| Behavior | 0.003 | 0.003 | 0.14 | NA | 0.28 | NA | 1.30 | 0.82 | 2.10 |
| Learning | 0.002 | 0.003 | 0.04 | 0.42 | 0.08 | NA | 1.58 | 0.94 | 2.64 |
| Speech/language | 0.02 | 0.01 | 1.0E-05 | 1.0E-04 | 1.8E-05 | 1.8E-04 | 1.50 | 1.24 | 1.83 |
| Multiple features | 0.06 | 0.05 | 8.8E-10 | 8.8E-09 | 1.7E-09 | 1.7E-08 | 1.38 | 1.24 | 1.53 |
| Rare CNVs | (n=4,705) | (n=2,652) |  |  |  |  |  |  |  |
| ID/DD no comorbidities | 0.43 | 0.41 | 0.04 | 0.36 | 0.07 | NA | 1.09 | 0.99 | 1.21 |
| Autism | 0.06 | 0.03 | 1.0E-09 | 1.0E-08 | 1.6E-09 | 1.6E-08 | 2.15 | 1.65 | 2.84 |
| OHI | 0.42 | 0.48 | 5.1E-08 | 5.1E-07 | 9.6E-08 | 9.6E-07 | 1.30 | 1.18 | 1.43 |
| Hearing | 0.003 | 0.004 | 0.35 | NA | 0.67 | NA | 1.27 | 0.50 | 3.07 |
| Vision | 0.001 | 0.001 | 0.63 | NA | 1.00 | NA | 1.13 | 0.16 | 12.47 |
| Psychiatric | 0.001 | 0.0004 | 0.41 | NA | 0.66 | NA | 2.26 | 0.22 | 111.07 |
| Behavior | 0.003 | 0.002 | 0.51 | NA | 1.00 | NA | 1.13 | 0.39 | 3.67 |
| Learning | 0.003 | 0.005 | 0.19 | NA | 0.31 | NA | 1.52 | 0.64 | 3.55 |
| Speech/language | 0.02 | 0.02 | 0.03 | 0.33 | 0.06 | NA | 1.42 | 0.98 | 2.09 |
| Multiple features | 0.06 | 0.06 | 0.10 | NA | 0.19 | NA | 1.15 | 0.93 | 1.42 |

Comparisons of the frequency of specific comorbid features in males and females with ID/DD in the clinical and rare CNV cohorts. **A graphical representation of ID/DD with and without comorbidities is shown in Figure 2B.** Fisher’s exact test was used for statistical analysis. Bonferroni correction was applied (corrected p-values) by multiplying p-values by 10, the number of tests performed in each cohort.

## Table S6: Frequency of comorbid specific OHI features among males and females with autism in the study cohort

| Clinical | Frequency in males (n=482) | Frequency in females (n=137) | 1 tail p-value | 1 tail corrected | 2 tail p-value | 2 tail corrected | Odds ratio | Lower 95% CI | Upper 95% CI |
| --- | --- | --- | --- | --- | --- | --- | --- | --- | --- |
| Epilepsy | 0.26 | 0.36 | 0.01 | 0.10 | 0.02 | 0.16 | 1.64 | 1.07 | 2.50 |
| Neurological disorder | 0.04 | 0.03 | 0.36 | NA | 0.62 | NA | 1.44 | 0.47 | 5.89 |
| Growth retardation | 0.06 | 0.06 | 0.49 | NA | 1.00 | NA | 1.11 | 0.48 | 2.86 |
| Kidney malformation | 0 | 0 | 1.00 | NA | 1.00 | NA | Inf | 0 | Inf |
| Cardiac abnormality | 0.02 | 0.01 | 0.48 | NA | 1.00 | NA | 1.43 | 0.30 | 13.57 |
| Other congenital anomaly | 0.12 | 0.09 | 0.28 | NA | 0.54 | NA | 1.28 | 0.66 | 2.63 |
| Dysmorphic features | 0.23 | 0.20 | 0.23 | NA | 0.42 | NA | 1.23 | 0.76 | 2.06 |
| Attention deficit disorders | 0.12 | 0.09 | 0.20 | NA | 0.36 | NA | 1.40 | 0.71 | 2.95 |
| Multiple features | 0.15 | 0.15 | 0.45 | NA | 0.79 | NA | 1.07 | 0.59 | 1.85 |
| Rare CNVs | (n=156) | (n=36) |  |  |  |  |  |  |  |
| Epilepsy | 0.23 | 0.28 | 0.35 | NA | 0.52 | NA | 1.28 | 0.50 | 3.07 |
| Neurological disorder | 0.006 | 0 | 0.81 | NA | 1.00 | NA | Inf | 5.9E-03 | Inf |
| Growth retardation | 0.08 | 0.08 | 0.56 | NA | 1.00 | NA | 1.09 | 0.19 | 4.36 |
| Kidney malformation | 0 | 0 | 1.00 | NA | 1.00 | NA | Inf | 0 | Inf |
| Cardiac abnormality | 0.05 | 0.03 | 0.47 | NA | 1.00 | NA | 1.89 | 0.24 | 86.29 |
| Other congenital anomaly | 0.13 | 0.17 | 0.35 | NA | 0.59 | NA | 1.36 | 0.41 | 3.91 |
| Dysmorphic features | 0.19 | 0.22 | 0.42 | NA | 0.65 | NA | 1.20 | 0.43 | 3.06 |
| Attention deficit disorders | 0.16 | 0.03 | 0.02 | 0.21 | 0.03 | 0.30 | 6.64 | 1.01 | 281.64 |
| Multiple features | 0.15 | 0.19 | 0.35 | NA | 0.62 | NA | 1.33 | 0.44 | 3.57 |

Comparisons of the frequency of each OHI feature in males and females with autism in the clinical and rare CNV cohorts. **A graphical representation of these features is shown in Figure S2.** Fisher’s exact test was used for statistical analysis. Bonferroni correction was applied (corrected p-values) by multiplying p-values by 9, the number of tests performed in each cohort.

## Table S7: Frequency of comorbid specific OHI features among males and females with ID/DD in the study cohort

| Clinical | Frequency in males (n=7,286) | Frequency in females (n=5,770) | 1 tail p-value | 1 tail corrected | 2 tail p-value | 2 tail corrected | Odds ratio | Lower 95% CI | Upper 95% CI |
| --- | --- | --- | --- | --- | --- | --- | --- | --- | --- |
| Epilepsy | 0.12 | 0.13 | 9.0E-03 | 0.08 | 0.02 | 0.16 | 1.14 | 1.02 | 1.27 |
| Neurological disorder | 0.02 | 0.02 | 0.49 | NA | 0.95 | NA | 1.01 | 0.80 | 1.27 |
| Growth retardation | 0.08 | 0.08 | 0.51 | NA | 1.00 | NA | 1.00 | 0.88 | 1.14 |
| Kidney malformation | 0.007 | 0.007 | 0.44 | NA | 0.83 | NA | 1.06 | 0.68 | 1.65 |
| Cardiac abnormality | 0.008 | 0.01 | 0.16 | NA | 0.30 | NA | 1.22 | 0.83 | 1.79 |
| Other congenital anomaly | 0.05 | 0.04 | 0.16 | NA | 0.30 | NA | 1.09 | 0.92 | 1.30 |
| Dysmorphic features | 0.46 | 0.44 | 4.2E-03 | 0.04 | 8.4E-03 | 0.08 | 1.10 | 1.02 | 1.18 |
| Attention deficit disorders | 0.01 | 0.006 | 5.7E-04 | 5.1E-03 | 8.8E-04 | 7.9E-03 | 1.95 | 1.29 | 3.03 |
| Multiple features | 0.24 | 0.26 | 0.01 | 0.12 | 0.03 | 0.24 | 1.10 | 1.01 | 1.19 |
| Rare CNVs | (n=1,962) | (n=1,277) |  |  |  |  |  |  |  |
| Epilepsy | 0.12 | 0.14 | 0.05 | 0.41 | 0.08 | NA | 1.21 | 0.97 | 1.49 |
| Neurological disorder | 0.02 | 0.03 | 0.10 | NA | 0.17 | NA | 1.37 | 0.86 | 2.18 |
| Growth retardation | 0.09 | 0.09 | 0.38 | NA | 0.75 | NA | 1.05 | 0.81 | 1.36 |
| Kidney malformation | 0.005 | 0.005 | 0.58 | NA | 1.00 | NA | 1.02 | 0.30 | 3.23 |
| Cardiac abnormality | 0.008 | 0.007 | 0.45 | NA | 0.84 | NA | 1.16 | 0.48 | 2.98 |
| Other congenital anomaly | 0.07 | 0.06 | 0.48 | NA | 0.94 | NA | 1.02 | 0.76 | 1.37 |
| Dysmorphic features | 0.42 | 0.36 | 1.0E-03 | 9.2E-03 | 2.0E-03 | 0.02 | 1.26 | 1.09 | 1.46 |
| Attention deficit disorders | 0.01 | 0.01 | 0.51 | NA | 0.87 | NA | 1.05 | 0.50 | 2.12 |
| Multiple features | 0.26 | 0.29 | 0.03 | 0.25 | 0.05 | NA | 1.17 | 1.00 | 1.37 |

Comparisons of the frequency of each OHI feature in males and females with ID/DD in the clinical and rare CNV cohorts. **A graphical representation of these features is shown in Figure S2.** Fisher’s exact test was used for statistical analysis. Bonferroni’s correction was applied (corrected p-values) by multiplying p-values by 9, the number of tests performed in each cohort.

## **Table S8: Frequency of comorbid features in males and females carrying CNVs associated with genomic disorders**

| CNV | Male Frequency | Male total | Female Frequency | Female total | 1-tailed p-value | 1-tail corrected | 2-tail p-value | 2-tail corrected | Odds ratio | Lower CI | Upper CI |
| --- | --- | --- | --- | --- | --- | --- | --- | --- | --- | --- | --- |
| 15q11.2_del | 63.79 | 58 | 55.26 | 38 | 0.27 | NA | 0.52 | NA | 1.42 | 0.57 | 3.56 |
| 15q13.3_del | 36.00 | 25 | 45.00 | 20 | 0.38 | NA | 0.56 | NA | 1.44 | 0.37 | 5.69 |
| 15q13.3_smalldup | 72.09 | 43 | 54.55 | 22 | 0.13 | NA | 0.18 | NA | 2.13 | 0.64 | 7.14 |
| 16p11.2_del | 46.67 | 45 | 65.52 | 29 | 0.09 | NA | 0.15 | NA | 2.15 | 0.75 | 6.43 |
| 16p11.2_dup | 55.17 | 29 | 62.50 | 16 | 0.44 | NA | 0.76 | NA | 1.35 | 0.33 | 5.81 |
| 16p13.11_dup | 64.00 | 25 | 53.85 | 26 | 0.33 | NA | 0.57 | NA | 1.51 | 0.43 | 5.45 |
| 17q21.31_del | 64.29 | 14 | 80.00 | 15 | 0.30 | NA | 0.43 | NA | 2.16 | 0.32 | 17.76 |
| 1p36_del | 63.16 | 19 | 80.00 | 25 | 0.18 | NA | 0.31 | NA | 2.29 | 0.50 | 11.44 |
| 1q21.1_del | 80.00 | 30 | 52.38 | 21 | 0.04 | 0.71 | 0.06 | NA | 3.54 | 0.90 | 15.26 |
| 1q21.1_dup | 62.96 | 27 | 60.00 | 20 | 0.54 | NA | 1.00 | NA | 1.13 | 0.29 | 4.34 |
| 22q11.2_DGSdel | 68.57 | 35 | 66.67 | 30 | 0.54 | NA | 1.00 | NA | 1.09 | 0.34 | 3.50 |
| 22q11.2_dup | 73.53 | 34 | 53.85 | 13 | 0.17 | NA | 0.29 | NA | 2.33 | 0.50 | 10.87 |
| 22q13_del | 54.55 | 11 | 52.94 | 34 | 0.60 | NA | 1.00 | NA | 1.07 | 0.22 | 5.34 |
| PWS_AS | 40.00 | 15 | 21.43 | 14 | 0.25 | NA | 0.43 | NA | 2.37 | 0.37 | 18.95 |
| PWS_dup | 61.54 | 26 | 28.57 | 21 | 0.02 | 0.46 | 0.04 | 0.75 | 3.88 | 1.01 | 16.70 |
| Smith-Magenis | 60.00 | 10 | 61.54 | 13 | 0.64 | NA | 1.00 | NA | 1.06 | 0.14 | 7.72 |
| Williams | 70.59 | 17 | 57.69 | 26 | 0.30 | NA | 0.52 | NA | 1.74 | 0.41 | 8.24 |
| 16p12.1_del | 50.00 | 20 | 55.56 | 9 | 0.55 | NA | 1.00 | NA | 1.24 | 0.20 | 8.30 |
| 16p13.11_del | 53.33 | 15 | 75.00 | 8 | 0.29 | NA | 0.40 | NA | 2.52 | 0.31 | 33.55 |

This table shows the comparisons of the frequency of comorbid features in males and females with ID/DD. Only CNVs with at least 10 males and 10 females were considered in this analysis (note: n<10 for females in 16p12.1 del and 16p13.11 del). Fisher’s exact test was used for statistical analysis. Bonferroni correction was applied (corrected p-values) by multiplying p-values by 19, the number of tests performed. **Representative deletions, duplications, and syndromic disorders from this table were used to generate Figure 2C.**

## Table S9: Ratio of males to females within the autism and the IDDD cohorts according to specific comorbidities

| **Individuals with autism** | **Male (%)** | **Female (%)** | **Ratio (Male:Female)** | **Sample size (n)** |
| --- | --- | --- | --- | --- |
| Psychiatric disorders | 89 | 11 | 8:1 | 9 |
| Other CNS malformations | 83 | 17 | 5:1 | 24 |
| Attention deficit disorders | 83 | 17 | 4.8:1 | 69 |
| Other congenital malformations | 81 | 19 | 4.2:1 | 260 |
| Autism (no comorbidities) | 81 | 19 | 4.2:1 | 2868 |
| ID/DD | 76 | 24 | 3.2:1 | 1376 |
| Speech and language disabilities | 72 | 28 | 2.6:1 | 25 |
| Epilepsy | 71 | 29 | 2.5:1 | 175 |
| **Individuals with ID/DD** |  |  |  |  |
| Autism | 76 | 24 | 3.2:1 | 1376 |
| Attention deficit disorders | 71 | 29 | 2.5:1 | 114 |
| Speech and language and learning | 67 | 33 | 2:1 | 577 |
| Behavior | 66 | 34 | 1.9:1 | 88 |
| ID/DD (no comorbidities) | 61 | 39 | 1.6:1 | 11651 |
| Psychiatric | 59 | 41 | 1.5:1 | 27 |
| Other congenital malformations | 57 | 43 | 1.3:1 | 7801 |
| Other CNS malformations | 56 | 44 | 1.3:1 | 315 |
| Epilepsy | 53 | 47 | 1.1:1 | 1583 |

A graphical representation of the data in this table is shown in Figure 3.

## Table S10: Ratio of males to females in individuals (with autism or ID/DD) carrying specific CNVs

| **Individuals with autism** | **Male (%)** | **Female (%)** | **Ratio (Male:Female)** | **Sample size (n)** |
| --- | --- | --- | --- | --- |
| 15q11.2 deletion | 62 | 38 | 1.6:1 | 13 |
| 15q13.3 deletion | 57 | 43 | 1.3:1 | 14 |
| 16p11.2 deletion | 56 | 44 | 1.3:1 | 16 |
| 16p11.2 duplication | 67 | 33 | 2:1 | 9 |
| 16p13.11 duplication | 67 | 33 | 2:1 | 12 |
| 1q21.1 duplication | 75 | 25 | 3:1 | 16 |
| 22q11.2 deletion | 67 | 33 | 2:1 | 6 |
| 22q11.2 duplication | 89 | 11 | 8:1 | 9 |
| 22q13 deletion | 63 | 38 | 1.7:1 | 8 |
| PWS duplications | 70 | 30 | 2.3:1 | 10 |
| **Individuals with ID/DD** |  |  |  |  |
| 15q11.2 deletion | 60 | 40 | 1.5:1 | 96 |
| 15q13.3 deletion | 56 | 44 | 1.3:1 | 45 |
| 16p11.2 deletion | 61 | 39 | 1.6:1 | 74 |
| 16p11.2 duplication | 64 | 36 | 1.8:1 | 45 |
| 16p13.11 duplication | 49 | 51 | 1:1 | 51 |
| 1q21.1 duplication | 57 | 43 | 1.4:1 | 47 |
| 22q11.2 deletion | 54 | 46 | 1.2:1 | 65 |
| 22q11.2_duplication | 72 | 28 | 2.6:1 | 47 |
| 22q13 deletion | 24 | 76 | 0.3:1 | 45 |
| PWS duplication | 55 | 45 | 1.2:1 | 47 |

A representative list of CNVs with sample size ≥5 is shown. **A graphical representation of the data in this table is shown in Figure 4.**

## Table S11: Comparison of rare CNV load for specific combination of comorbid features in autism and ID/DD

| Cohort 1 | Cohort 1 frequency | Cohort 1 total | Cohort 2 | Cohort 2 frequency | Cohort 2 total | One tail p-value | Odds ratio | Lower CI | Upper CI |
| --- | --- | --- | --- | --- | --- | --- | --- | --- | --- |
| 1 Mb |  |  |  |  |  |  |  |  |  |
| Autism male | 0.12 | 833 | Autism female | 0.18 | 183 | 0.01 | 1.69 | 1.06 | 2.64 |
| Autism+ID-DD male | 0.14 | 457 | Autism+ID-DD female | 0.25 | 145 | 3.5E-03 | 1.95 | 1.20 | 3.16 |
| Autism+epilepsy male | 0.15 | 105 | Autism+epilepsy female | 0.26 | 47 | 0.10 | 1.90 | 0.74 | 4.79 |
| ID-DD+epilepsy male | 0.20 | 415 | ID-DD+epilepsy female | 0.31 | 316 | 6.2E-04 | 1.77 | 1.24 | 2.52 |
| Autism no comorbidities male | 0.11 | 658 | Autism no comorbidties female | 0.18 | 141 | 0.02 | 1.73 | 1.01 | 2.89 |
| Autism+comorbidities male | 0.14 | 632 | Autism+comorbidties female | 0.24 | 187 | 2.0E-03 | 1.88 | 1.22 | 2.86 |
| ID-DD no comorbidites male | 0.18 | 2021 | ID-DD no comorbidities female | 0.30 | 1081 | 1.3E-14 | 1.98 | 1.66 | 2.36 |
| ID-DD+comorbidities male | 0.22 | 2684 | ID-DD+comorbidities female | 0.32 | 1571 | 1.1E-14 | 1.73 | 1.50 | 2.00 |
| Autism no comorbidities male | 0.11 | 658 | Autism+comorbidities male | 0.14 | 632 | 0.06 | 1.31 | 0.93 | 1.86 |
| Autism no comorbidities female | 0.18 | 141 | Autism+comorbidities female | 0.24 | 187 | 0.13 | 1.43 | 0.80 | 2.59 |
| ID-DD no comorbidities male | 0.18 | 2021 | ID-DD+comorbidities male | 0.22 | 2684 | 2.7E-04 | 1.30 | 1.12 | 1.51 |
| ID-DD no comorbidities female | 0.30 | 1081 | ID-DD+comorbidities female | 0.32 | 1571 | 0.07 | 1.14 | 0.96 | 1.35 |
| 1.5 Mb |  |  |  |  |  |  |  |  |  |
| Autism male | 0.06 | 833 | Autism female | 0.10 | 183 | 0.02 | 1.85 | 1.00 | 3.30 |
| Autism+ID-DD male | 0.11 | 457 | Autism+ID-DD female | 0.15 | 145 | 0.13 | 1.42 | 0.79 | 2.50 |
| Autism+epilepsy male | 0.09 | 105 | Autism+epilepsy female | 0.15 | 47 | 0.19 | 1.86 | 0.55 | 6.06 |
| ID-DD+epilepsy male | 0.15 | 415 | ID-DD+epilepsy female | 0.24 | 316 | 1.7E-03 | 1.77 | 1.20 | 2.60 |
| Autism no comorbidities male | 0.06 | 658 | Autism no comorbidties female | 0.09 | 141 | 0.08 | 1.70 | 0.81 | 3.39 |
| Autism+comorbidities male | 0.10 | 632 | Autism+comorbidties female | 0.15 | 187 | 0.04 | 1.59 | 0.95 | 2.62 |
| ID-DD no comorbidites male | 0.13 | 2021 | ID-DD no comorbidities female | 0.22 | 1081 | 2.1E-11 | 1.94 | 1.59 | 2.37 |
| ID-DD+comorbidities male | 0.16 | 2684 | ID-DD+comorbidities female | 0.25 | 1571 | 2.5E-11 | 1.68 | 1.44 | 1.96 |
| Autism no comorbidities male | 0.06 | 658 | Autism+comorbidities male | 0.10 | 632 | 2.4E-03 | 1.86 | 1.20 | 2.92 |
| Autism no comorbidities female | 0.09 | 141 | Autism+comorbidities female | 0.15 | 187 | 0.08 | 1.73 | 0.83 | 3.80 |
| ID-DD no comorbidities male | 0.13 | 2021 | ID-DD+comorbidities male | 0.16 | 2684 | 1.3E-04 | 1.37 | 1.15 | 1.62 |
| ID-DD no comorbidities female | 0.22 | 1081 | ID-DD+comorbidities female | 0.25 | 1571 | 0.04 | 1.18 | 0.98 | 1.43 |
| 2 Mb |  |  |  |  |  |  |  |  |  |
| Autism male | 0.04 | 833 | Autism female | 0.09 | 183 | 0.02 | 2.12 | 1.07 | 4.03 |
| Autism+ID-DD male | 0.09 | 457 | Autism+ID-DD female | 0.14 | 145 | 0.04 | 1.72 | 0.93 | 3.10 |
| Autism+epilepsy male | 0.08 | 105 | Autism+epilepsy female | 0.15 | 47 | 0.14 | 2.11 | 0.61 | 7.17 |
| ID-DD+epilepsy male | 0.12 | 415 | ID-DD+epilepsy female | 0.19 | 316 | 8.5E-03 | 1.67 | 1.09 | 2.57 |
| Autism no comorbidities male | 0.04 | 658 | Autism no comorbidties female | 0.08 | 141 | 0.07 | 1.90 | 0.83 | 4.06 |
| Autism+comorbidities male | 0.08 | 632 | Autism+comorbidties female | 0.14 | 187 | 9.7E-03 | 1.92 | 1.11 | 3.26 |
| ID-DD no comorbidites male | 0.11 | 2021 | ID-DD no comorbidities female | 0.19 | 1081 | 1.2E-09 | 1.90 | 1.54 | 2.35 |
| ID-DD+comorbidities male | 0.14 | 2684 | ID-DD+comorbidities female | 0.21 | 1571 | 8.4E-10 | 1.67 | 1.41 | 1.97 |
| Autism no comorbidities male | 0.04 | 658 | Autism+comorbidities male | 0.08 | 632 | 5.5E-03 | 1.89 | 1.15 | 3.17 |
| Autism no comorbidities female | 0.08 | 141 | Autism+comorbidities female | 0.14 | 187 | 0.06 | 1.90 | 0.87 | 4.44 |
| ID-DD no comorbidities male | 0.11 | 2021 | ID-DD+comorbidities male | 0.14 | 2684 | 2.3E-03 | 1.30 | 1.08 | 1.56 |
| ID-DD no comorbidities female | 0.19 | 1081 | ID-DD+comorbidities female | 0.21 | 1571 | 0.10 | 1.14 | 0.93 | 1.39 |

Comparisons between the CNV burdens in different cohorts are shown in Figure 5. One-tailed Fisher’s exact test was used for comparisons.

## Table S12: Comparison of CNV burden between males and females ascertained for autism or controls in two independent cohort studies

| Cohort 1 | Cohort 1 frequency | Cohort 1 total | Cohort 2 | Cohort 2 frequency | Cohort 2 total | p-value | Odds ratio | Lower CI | Upper CI |
| --- | --- | --- | --- | --- | --- | --- | --- | --- | --- |
| 1 Mbp |  |  |  |  |  |  |  |  |  |
| MIND ASD (no ID) Female | 0.11 | 28 | MIND ASD (no ID) Male | 0.04 | 195 | 0.12 | 3.20 | 0.50 | 15.17 |
| Controls Female | 0.00 | 36 | Controls Male | 0.01 | 180 | 0.83 | Inf | 0.01 | Inf |
| Sanders ASD (no ID) Female | 0.06 | 95 | Sanders ASD (no ID) Male | 0.07 | 761 | 0.57 | 1.04 | 0.43 | 3.06 |
| Sanders ASD+ID Female | 0.11 | 53 | Sanders ASD+ID Male | 0.08 | 183 | 0.32 | 1.43 | 0.43 | 4.17 |
| 1.5 Mbp |  |  |  |  |  |  |  |  |  |
| MIND ASD (no ID) Female | 0.07 | 28 | MIND ASD (no ID) Male | 0.02 | 195 | 0.17 | 3.64 | 0.31 | 26.86 |
| Controls Female | 0.00 | 36 | Controls Male | 0.00 | 180 | 1.00 | 0.00 | 0.00 | Inf |
| Sanders ASD (no ID) Female | 0.03 | 95 | Sanders ASD (no ID) Male | 0.05 | 761 | 0.37 | 1.48 | 0.45 | 7.66 |
| Sanders ASD+ID Female | 0.11 | 53 | Sanders ASD+ID Male | 0.07 | 183 | 0.23 | 1.67 | 0.49 | 5.01 |
| 2 Mbp |  |  |  |  |  |  |  |  |  |
| MIND ASD (no ID) Female | 0.04 | 28 | MIND ASD (no ID) Male | 0.02 | 195 | 0.49 | 1.76 | 0.03 | 18.70 |
| Controls Female | 0.00 | 36 | Controls Male | 0.00 | 180 | 1.00 | 0.00 | 0.00 | Inf |
| Sanders ASD (no ID) Female | 0.02 | 95 | Sanders ASD (no ID) Male | 0.02 | 761 | 0.55 | 1.15 | 0.12 | 5.12 |
| Sanders ASD+ID Female | 0.08 | 53 | Sanders ASD+ID Male | 0.03 | 183 | 0.12 | 2.89 | 0.55 | 13.99 |

One-tailed Fisher’s exact test was used for comparisons.

# 2. Supplemental Figures

Figure S1: Age specific prevalence of ID/DD/MCA (left) and neuropsychiatric/behavioral features (right) within the clinical dataset. The results suggest that ID/DD/MCA phenotypes are more likely to manifest in the early childhood while neuropsychiatric and behavioral features are higher in older children. The same criteria/classifications were used for categorizing family history data in our analysis.

Figure S2: Frequency of comorbid features within the OHI category for clinical and rare CNV cohorts and for males and females with autism features **(A)** or ID/DD **(B)** within the clinical cohort, and (**C-D**) those within the rare CNV cohort. Raw counts and frequencies are presented in Tables S6 and S7.

Figure S3: Replication of CNV burden results in two independent cohort studies. (A) CNV datasets from the CHARGE study [[1](#_ENREF_1)] (see methods) (B) and (C) CNV datasets from the SSC study [[2](#_ENREF_2)]. Note that here, ASD=ASD with no ID/DD. Statistical analysis is given in Table S12 for (**A**) and (**B**). Mann Whitney test comparing males and females in (**C**) showed a significant difference, one-tailed p=0.009.


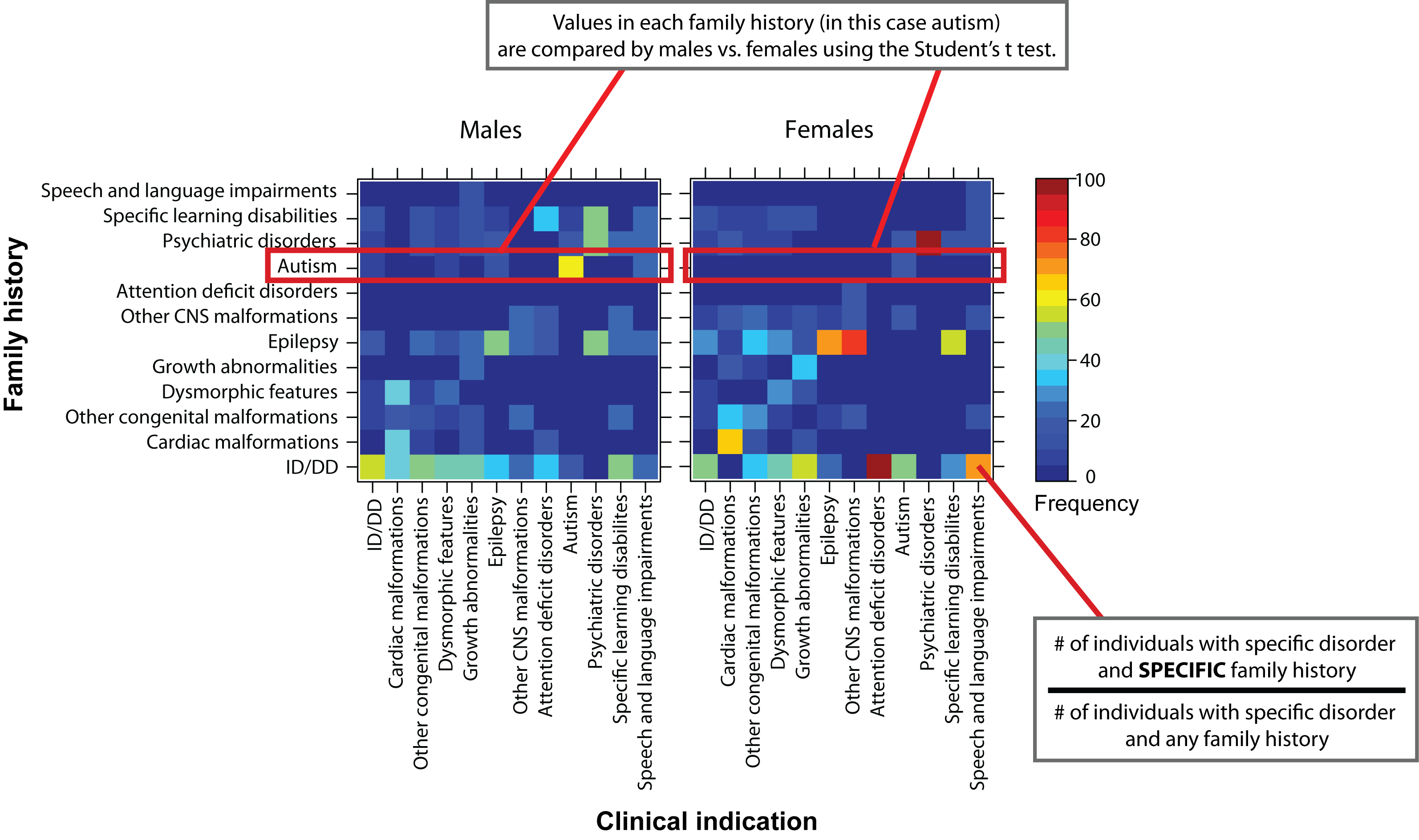


Figure S4: Explanation of the comparisons made in the family history matrices (Figure 6). Each cell used to make the Figure 6 level plot was generated by dividing the number of individuals with specific indication and a specific family history (numerator) by the number of individuals with the specific disorder and any family history (denominator). For each family history section, the values corresponding to males and females were compared by Student’s t test.

# REFERENCES

1. Girirajan S, Johnson RL, Tassone F, Balciuniene J, Katiyar N, Fox K et al. Global increases in both common and rare copy number load associated with autism. Hum Mol Genet. 2013;22(14):2870-80.

2. Sanders SJ, Ercan-Sencicek AG, Hus V, Luo R, Murtha MT, Moreno-De-Luca D et al. Multiple recurrent de novo CNVs, including duplications of the 7q11.23 Williams syndrome region, are strongly associated with autism. Neuron. 2011;70(5):863-85.
